# The effect of sensor-based exercise at home on functional performance associated with fall risk in older people – a comparison of two exergame interventions

**DOI:** 10.1186/s11556-015-0156-5

**Published:** 2015-11-30

**Authors:** Yves J. Gschwind, Daniel Schoene, Stephen R. Lord, Andreas Ejupi, Trinidad Valenzuela, Konstantin Aal, Ashley Woodbury, Kim Delbaere

**Affiliations:** NeuRA, UNSW, Randwick, Australia; Assistive Healthcare Information Technology Group, Austrian Institute of Technology, Vienna, Austria; Exercise Science Laboratory, School of Kinesiology, Faculty of Medicine, Universidad Finis Terrae, Santiago, Chile; Institute for Information Systems, University Siegen, Siegen, Germany

**Keywords:** Fall prevention, Aged, Stepping, Balance, Exergames, Cognition

## Abstract

**Background:**

There is good evidence that balance challenging exercises can reduce falls in older people. However, older people often find it difficult to incorporate such programs in their daily life. Videogame technology has been proposed to promote enjoyable, balance-challenging exercise. As part of a larger analysis, we compared feasibility and efficacy of two exergame interventions: step-mat-training (SMT) and Microsoft-Kinect® (KIN) exergames.

**Methods:**

148 community-dwelling people, aged 65+ years participated in two exergame studies in Sydney, Australia (KIN: *n* = 57, SMT: *n* = 91). Both interventions were delivered as unsupervised exercise programs in participants’ homes for 16 weeks. Assessment measures included overall physiological fall risk, muscle strength, finger-press reaction time, proprioception, vision, balance and executive functioning.

**Results:**

For participants allocated to the intervention arms, the median time played each week was 17 min (IQR 32) for KIN and 48 min (IQR 94) for SMT. Compared to the control group, SMT participants improved their fall risk score (*p* = 0.036), proprioception (*p* = 0.015), reaction time (*p* = 0.003), sit-to-stand performance (*p* = 0.011) and executive functioning (*p* = 0.001), while KIN participants improved their muscle strength (*p* = 0.032) and vision (*p* = 0.010), and showed a trend towards improved fall risk scores (*p* = 0.057).

**Conclusions:**

The findings suggest that it is feasible for older people to conduct an unsupervised exercise program at home using exergames. Both interventions reduced fall risk and SMT additionally improved specific cognitive functions. However, further refinement of the systems is required to improve adherence and maximise the benefits of exergames to deliver fall prevention programs in older people’s homes.

**Trial registrations:**

ACTRN12613000671763 (Step Mat Training RCT)

ACTRN12614000096651 (MS Kinect RCT)

## Background

Unstable balance, lower extremity muscle weakness and impaired cognitive functions (executive functions, processing speed) are important risk factors for falls in older adults [1]. There is strong systematic review evidence that exercise interventions including high intensity balance training (i.e., exercises that reduce the base of support, minimize upper limb support and include weight shifting) are most effective in preventing falls in older people [2]. To our knowledge, there is no study investigating the impact of cognitive training on falls, but robust evidence suggests that physical exercise may improve cognition [3]. One study found that an exercise program known to reduce falls led to improved response inhibition in addition to physical improvements of balance and muscle strength [4].

It has been suggested that in order to reduce falls, older people should engage in at least 2 h of strength and balance training per week over an extended period of time [5]. In order to reach this exercise training dose, without overwhelming specialist services, older people need to perform at least part of the training at home. However, incorporating a new habitual exercise regimen is challenging for many older people due to poor exercise tolerance and enjoyment [6]. Furthermore, additional challenges arise when conducting unsupervised exercise programs in home settings where factors such as adherence, progression, safety, quality and dosage are harder to control compared to exercise under supervision of an exercise professional.

Recently, videogame technology has been increasingly used to deliver exercise programs (often referred to as “exergames”) to address this problem. Exergames offer engaging video games instead of repetitive conventional exercises, provide instant performance feedback and unlock levels of difficulty according to the individual’s performance. Accordingly, exergames might enhance motivation and replace or complement more resource-demanding traditional approaches of exercise delivery [7, 8]. However, while a plethora of such exergames is available, evidence-based programs in a home setting remain sparse. A recent systematic review of 37 studies concluded that there is preliminary evidence that exergame interventions can improve physical and cognitive fall risk factors in older people [9]. It further suggested that these exergame interventions are of equivalent efficacy in reducing fall risk as traditional training programs. However, the review also found that the methodological quality of the included studies was often poor, sample sizes were mostly small, and only three [10–12] of the 37 studies were carried out unsupervised by older people at home. Clearly, more research is required before exergames could be recommended as a fall prevention strategy.

A wide variety of exergames has been explored which can provide challenging balance exercises. Two promising exergame interventions that require further investigation are step mat training (SMT) and Microsoft Kinect® (KIN) exergames. As part of the iStoppFalls research program [13], we compared the feasibility and efficacy of SMT (trial registration ACTRN12613000671763) and KIN strength and balance exercises (trial registration ACTRN12614000096651). Specifically, we compared the two home-based interventions that took place in Sydney, Australia, with respect to adherence and efficacy in relation to functional performance outcomes in older people. SMT was played by using a step mat as input device on which participants had to repetitively step in multiple directions under varying cognitive load that required attention, executive control and fast processing. KIN player’s movements were mirrored by an avatar in a virtual environment on a television (TV). KIN games were specifically designed to improve balance and lower extremity strength. Based on the exergame components and principle of task specificity in exercise training, we hypothesised that the SMT would have a greater effect on improving cognitive functioning whereas KIN would have greater effects on muscle strength and balance.

## Methods

### Participants

The sample comprised 148 participants from the SureStep study and the Australian arm of the iStoppFalls study: (KIN: *n* = 57, SMT: *n* = 91). Participants were recruited from retirement villages and the community in Sydney, Australia. Individuals were eligible if they were: 1) aged 65 years or older, 2) living independently, 3) able to walk with or without a walking aid. For the SMT randomised controlled trial (RCT), potential participants were 70 years or older, and were excluded if they were not able to step unassisted on a step pad (step size 25–30 cm), or had severe lower extremity pain that prevented them from step training. For the KIN RCT, participants had to be able to watch TV with or without their glasses from 3 m distance. General exclusion criteria were: 1) major cognitive impairments (Mini-Cog score <3) [14], 2) medical conditions preventing regular exercise (i.e., neurodegenerative disease, cardiovascular disease, psychiatric disorder), 3) colour blindness and 4) insufficient language skills to understand the study procedures. All participants gave written informed consent prior to inclusion. Ethical approval was given by the Human Research Ethics Committee of the University of New South Wales.

### Randomization

In both studies, eligible participants were randomised (ratio 1:1) by permuted block-randomisation using computer-generated random numbers. Block size was random and ranged from four to eight. Participants living in the same household were treated as one unit and randomised into the same block.

### Intervention design

The interventions were delivered as unsupervised exercise programs in participants’ homes. Both, the KIN and SMT systems are described in detail elsewhere [13, 15]. Both contained an input device to record exercise data, a computer to deliver the intervention tasks and store the data, and universal serial bus (USB) modems to provide remote access to the computers by research staff. Additionally, the participants’ TVs were used as projection screens for the exergames. During a 90 min introductory session the systems were installed in an appropriate location within participants’ homes. Participants were provided with teaching manuals and instructed on how to use the system and play the games safely. Exercise data (i.e., frequency, duration, game level) for both 16-week interventions were stored on the systems’ computers. Participants had continuous access to their performance scores on the physical tests (KIN) and games (SMT, KIN). To discuss any issues related to system use and exercises, participants were contacted by monthly phone calls. In case of technical issues, participants could call the research team for phone support, or to request additional home visits.

The KIN exergames consisted of three specifically developed balance exergames (i.e., walking, stepping, weight shifting) based on the Weight-bearing Exercise for Better Balance (WEBB) program (www.webb.org.au), and five strength exercises (i.e., knee extension/flexion, hip abduction, calf/toe raises) based on the Otago Exercise Program [16, 17] targeting fall risk factors such as unstable balance and muscle weakness of the lower limbs. Participants’ movements were recorded by the Kinect (3D depth sensor) and displayed as an avatar on screen. Participants were encouraged to perform 120 min of balance exergames per week and 60 min of strength exercises per week. Progression of training intensity was possible by increasing the level of difficulty (i.e., by including secondary cognitive memory tasks (dual-tasking) for balance exergames) and by increasing the number of repetitions and sets or ankle cuff weights (1 kg, 2 kg or 3 kg) for strength exercises.

The SMT comprised of exergames each targeting specific cognitive functions associated with fall risk and stepping: a modified StepMania game (divided attention, inhibition and processing speed; timed and coordinated stepping according to arrows on screen that differed in their direction and drift speed), Stepper (processing speed, selective attention; rapid stepping in four directions), Trail-stepping (visual attention, set-shifting; stepping to connect numbers and letters) and Tetris-stepping (visuo-spatial skills, problem-solving; stepping used to rotate and control blocks of different shapes). Participants were able to play the exergames by stepping onto six arrows (pointing to the front, side and back) on a pressure-sensitive electronic mat. Participants were instructed to perform a minimum of three 20 min sessions per week each including all four exergames. Progression was possible by the selection of more difficult exergame levels with higher cognitive load, stepping speed and complexity.

The control group was provided with an educational booklet about evidence-based health and fall prevention advice [18], and asked to remain their usual activities for the duration of the study period.

### Outcomes

Participants were assessed at baseline and at the end of the intervention period (16 weeks) by blinded assessors. Socio-demographic and medical information was collected by self-report questionnaires. The 12-item World Health Organization Disability Assessment Schedule (WHODAS) 2.0 was used as a generic assessment instrument of health and disability. Participants reported their level of impairment for several instrumental activities of daily living on a five-point Likert scale (www.who.int/classifications/icf/whodasii/en/). The Mini-Cog was used as screening test to detect major cognitive impairments. It consists of two tasks, delayed recall of three items and the clock drawing test as a measure of executive functioning and visuo-spatial ability [2]. Anthropometric measures were obtained during the assessments.

The following assessment measures were included in the comparison analyses. Fall risk was assessed by the Physiological Profile Assessment (PPA) which includes five sensorimotor tests [19]: (i) the Melbourne Edge Test of visual contrast sensitivity; (ii) a lower limb-matching task to assess proprioception, with errors in degrees recorded using a protractor marked on a vertical clear acrylic sheet placed between the legs; (iii) quadriceps strength measured isometrically in the dominant leg with participants seated with the hip and knee flexed 90°; (iv) finger-press reaction time assessed using a light as stimulus and a finger-press as the response; (v) postural sway measured using a sway meter recording displacements of the body at the level of the pelvis with participants standing on a foam rubber mat with eyes open. Weighted contributions from these measures have been shown to discriminate between older fallers and non-fallers with an accuracy of up to 75 % [20].

Functional mobility was assessed using the timed up and go test (TUG) [21], and the five times sit-to stand test (STS) [22]. For the TUG, participants were asked to stand up from a chair, walk to a three meter mark at their self-selected usual pace, return, and sit down in the chair again. For the STS, participants were instructed to cross their arms over the abdomen, and rise from a chair for five times as quickly as possible. For both tasks, the time to complete was recorded in seconds.

Cognitive performance was assessed using the Attention Network Test (ANT) [23], the Victoria Stroop Test (VST) [24], and the Digit span backward (DSB) Test [25]. The ANT requires participants to determine whether a central arrow points to the left or right, and was used to quantify processing efficiency within three attentional networks (alerting, orienting, executive attention) by measuring how response times are influenced by alerting cues, spatial cues, and flankers. The VST requires participants to state a colour under three conditions, while supressing habitual responses related to the conditions. The VST was used to assess executive control by response inhibition. For this secondary analysis we only used the following VST outcomes: time to complete and number of errors made during the colour-word interference task (condition 3) and the efficiency score of inhibition calculated as the ratio of colour-word interference and colour only tasks (condition 3/condition 1). The DSB requires participants to repeat sequences of numbers with increasing length (two to nine digits) in reverse order as stated by the investigator, and was used as a measure of working memory.

### Statistical analyses

Variables used in this comparison analysis were normally distributed with or without log-transformation. Differences between groups at baseline were analysed by analysis of variance (ANOVA) with Tukey’s post-hoc tests for pairwise comparisons for continuous variables and chi square tests for categorical data. Generalized linear modelling (GLM) was used to determine the intervention effects for both training modes and comparing it with the combined control group. Age and baseline performance of the variable under investigation were entered as covariates in all models. The alpha level was set at 5 %. Analyses were performed with SPSS version 22 for Windows (SPSS, Inc., Chicago, IL).

## Results

**Fig. 1:**
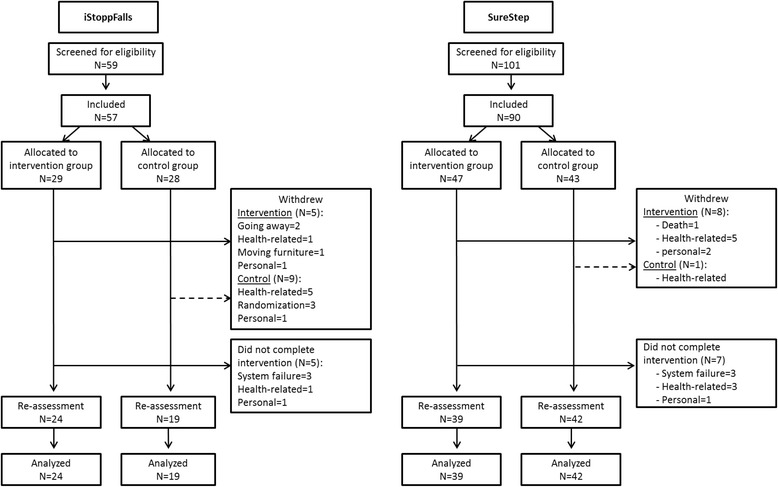
Study flow chart

Of the 148 people recruited (KIN = 57, SMT = 91); 76 were allocated to the intervention groups (KIN = 29, SMT = 47) and 72 to the control groups (KIN = 28, SMT = 44). Participant characteristics are summarised in Table 1. Participants across the two studies did not differ in demographic characteristics (Table 1). However, there were differences in some of the outcome measures at baseline (Table 2). At baseline, the KIN intervention group performed better than the SMT intervention group and combined control group in a number of measures: PPA overall score (F = 7.69, df = 2, *p* = 0.001), proprioception (F = 5.14, df = 2, *p* = 0.007), postural sway (F = 6.16, df = 2, *p* = 0.003), and DSB (F = 7.449, df = 2, *p* = .001). One-hundred and twenty-four participants were re-assessed after the completion of the training period (KIN = 43, IG = 24, CG = 19; SMT = 81, IG = 39, CG = 42) and included in the analyses using intention-to-treat principles. Figure 1 shows the flow of participants through the studies (Fig. 1).

**Table 1:** Participant characteristics (mean and standard deviation)

	KIN	SMT	Control	*p*-value
	(*n* = 24)	(*n* = 39)	(*n* = 61)	
Age (years)	80.1 (6.3)	82.5 (7.0)	80.2 (6.5)	0.195
Height (cm)	161.8 (7.7)	163.2 (9.7)	162.5 (10.1)	0.863
Weight (kg)	69.8 (12.3)	73.4 (18.3)	70.1 (13.9)	0.526
Medication use (n)	4.6 (3.0)	4.2 (3.0)	4.4 (3.0)	0.872
Comorbidities (n)	3.2 (1.5)	3.0 (1.6)	3.1 (1.8)	0.934
WHODAS (score)	16.8 (3.7)	16.4 (5.0)	16.2 (4.0)	0.822
Mini-Cog (score)	4.6 (0.7)	4.4 (0.8)	4.5 (0.7)	0.571
Gender (female)	62.5 %	69.2 %	65.6 %	0.853
Fallen past year (yes)	37.5 %	35.9 %	24.6 %	0.352
Use of walking aids (yes)	16.7 %	23.1 %	27.9 %	0.544

®KIN Microsoft-Kinect®, SMT step-mat-training, *WHODAS* World Health Organization Disability Assessment Schedule

**Table 2:** Group comparison of physical and cognitive outcomes at baseline (mean and standard deviation, SD)

	KIN	SMT	Control	
				*p*-value
PPA score	1.1 (0.8)	2.0 (0.9)	1.8 (1.1)	**0.001** ^*,**^
Contrast sensitivity score^b^	21.6 (1.9)	20.8 (2.2)	21.0 (2.6)	0.412
Proprioception (degree)^a^	1.8 (1.1)	2.9 (1.7)	2.6 (1.6)	**0.007** ^*,**^
Knee extension strength (kg)^b^	20.8 (9.4)	24.2 (10.3)	21.9 (8.6)	0.318
Standing balance (mm)^a^	261 (124)	377 (152)	379 (188)	**0.003** ^*,**^
Finger-press reaction time (ms)	240 (45)	259 (45)	247 (45)	0.225
Timed up & go (s)^a^	11.5 (3.5)	11.5 (3.1)	12.4 (3.7)	0.317
Sit-to-stand transfer (s)^a^	13.6 (4.8)	13.1 (3.8)	14.4 (4.9)	0.340
ANT alert (ms)^b^	34 (32)	34 (35)	39 (33)	0.789
ANT orient (ms)^b^	40 (43)	60 (43)	44 (45)	0.128
ANT conflict (ms)^a^	118 (67)	154 (120)	137 (74)	0.533
Stroop CW_incongruency - time (s)	68.3 (32.3)	65.0 (28.4)	75.5 (38.0)	0.314
Stroop CW_incongruency - errors^a^	4.5 (3.4)	6.1 (4.0)	6.2 (5.4)	0.270
Stroop efficiency (CW_incongruent/C)	2.0 (0.5)	2.2 (0.8)	2.4 (1.0)	0.147
Digit span backwards (length of sequence)^ab^	6.8 (2.6)	5.1 (1.0)	5.3 (1.4)	**0.001** ^*,**^

^a^Log transformed

^b^Higher values indicate better performance KIN Microsoft-Kinect®, SMT step-mat-training, *PPA* Physiological Profile Assessment, *ANT* Attentional Network Test, *CW* colour-word, *C* colour

^*^Post-hoc tests (Tukey) results indicate difference between KIN and Control

^**^Post-hoc tests (Tukey) results indicate difference between KIN and SMT

**Table 3:** Between-group intervention effects

	KIN (IG 1)		SMT (IG 2)		Control				
	Pre	Post	Pre	Post	Pre	Post	*p*-value	*p*-value	*p*-value
	Mean (SD)	Mean (SD)	Mean (SD)	Mean (SD)	Mean (SD)	Mean (SD)	IG1-IG2	IG1-CG	IG2-CG
PPA score	1.1 (0.8)	0.9 (1.1)	2.0 (0.9)	1.5 (0.8)	1.8 (1.1)	1.6 (0.9)	.866	*.057*	**.036**
Contrast sensitivity score^b^	21.6 (1.9)	23.0 (1.1)	20.8 (2.2)	21.4 (1.6)	21.0 (2.6)	21.9 (2.0)	**.003**	**.010**	.462
Proprioception (degree)^a^	1.8 (1.1)	1.8 (1.3)	2.9 (1.7)	2.0 (1.3)	2.6 (1.6)	2.5 (1.6)	.486	.192	**.015**
Knee extension strength (kg)^b^	20.8 (9.4)	26.2 (10.3)	24.2 (10.3)	25.8 (9.2)	21.9 (8.6)	23.8 (9.1)	.131	**.032**	.578
Standing balance (mm)^a^	261 (124)	263 (159)	377 (152)	332 (131)	379 (188)	337 (189)	.481	.431	.967
Finger-press reaction time (ms)	240 (45)	239 (49)	259 (45)	238 (40)	247 (45)	247 (41)	*.057*	.611	**.003**
Timed up & go (s)^a^	11.5 (3.5)	11.1 (3.3)	11.5 (3.1)	11.5 (2.6)	12.4 (3.7)	12.6 (4.4)	.298	.190	.848
Sit-to-stand transfer (s)^a^	13.6 (4.8)	12.1 (3.7)	13.1 (3.8)	11.4 (2.7)	14.4 (4.9)	13.3 (3.8)	.370	.197	**.011**
ANT alert (ms)^b^	34 (32)	45 (36)	34 (35)	35 (33)	39 (33)	37 (38)	.285	.243	.990
ANT orient (ms)^b^	40 (43)	47 (47)	60 (43)	57 (38)	44 (45)	57 (40)	.886	.375	.418
ANT conflict (ms)^a^	118 (67)	111 (46)	154 (120)	103 (41)	137 (74)	111 (46)	.196	.267	.720
Stroop time (s)	68.3 (32.3)	62.5 (28.3)	65.0 (28.4)	26.8 (21.0)	75.5 (38.0)	47.1 (33.1)	**<.001**	**<.001**	**.001**
Stroop errors^a^	4.5 (3.4)	3.9 (3.8)	6.1 (4.0)	4.7 (4.2)	6.2 (5.4)	5.6 (5.0)	.516	.437	*.085*
Stroop efficiency	2.0 (0.5)	1.9 (0.7)	2.2 (0.8)	1.9 (0.7)	2.4 (1.0)	2.2 (0.8)	.298	.589	*.054*
Digit span backwards^ab^	6.8 (2.6)	6.5 (2.6)	5.1 (1.0)	5.2 (1.0)	5.3 (1.4)	5.3 (1.4)	.634	.263	.473

^a^Log transformed

^b^Higher values indicate better performance IG inervention group, CG, control group, KIN Microsoft-Kinect®, SMT step-mat-training, *PPA* Physiological Profile Assessment, *ANT* Attentional Network Test, *CW* colour-word, *C* colour

Note: Significant p-values in bold, p-values between .1 and .05 in italics

For participants allocated to the intervention arms, the median exercise time for the KIN system was 4.5 (interquartile range, IQR 8.4) hours and 12.7 (IQR 25) hours for the SMT system during the 16 week intervention periods. The median time played each week was 17 min (IQR 32) for KIN and 48 min (IQR 94) for SMT. No adverse events associated with the intervention were reported for either study. However, technical difficulties impacted on training time.

Between-group differences (controlling for age and baseline performance) for the two interventions are presented in Table 3, compared to the combined control group and compared to each other. Compared to the control group, the KIN intervention group improved significantly in tests of contrast sensitivity and isometric knee extension strength and showed a trend towards improved PPA fall risk scores. A significant between-group difference in Stroop time was due to an improved performance in the control group compared to little change in the KIN intervention group. The SMT intervention group had significantly improved PPA fall risk scores at reassessment compared with the control group and showed significant improvements in the proprioception, finger-press reaction time, sit-to-stand and Stroop time as well as trends for improvements in the number of errors made and the efficiency score in the Stroop test. When comparing the two intervention groups, the following outcome measures were different at re-assessment: KIN participants had significantly better contrast sensitivity than SMT participants and SMT participants had a faster Stroop time than KIN training participants. There was also a trend indicating SMT participants had faster finger-press reaction times than KIN participants.

## Discussion

Our findings are partly in line with the hypotheses that SMT would have a greater effect on cognitive functioning and KIN training would have greater effects on strength and balance. SMT, with its strong motor-cognitive component, did improve cognitive processing measures (processing speed, inhibition) as well as proprioception, sit-to-stand times and overall physiological fall risk. The KIN training, with its specific strength and balance exercises, improved knee extension strength as well as visual contrast sensitivity. Both intervention groups reduced their overall physiological fall risk (as measured by the PPA) compared to the control group suggesting the interventions have potential efficacy for fall prevention. However, when comparing the two intervention groups directly, KIN participants improved more than SMT participants in the contrast sensitivity test and SMT participants improved more than KIN participants in the Stroop and finger-press reaction time tests.

Some effects of the two interventions require comment. The obtained findings reflect task-specific changes. First, the SMT included games that required fast central processing, dual-tasking and inhibiting irrelevant stimuli. Therefore, the improvement in finger-press simple reaction time in the SMT group suggests SMT-induced improvements in central processing and is consistent with a previous step training study [26]. Improvements in time and efficiency of the Stroop task may be underpinned by this faster processing. However, the reduced number of errors suggests additional improvements in selective attention and inhibitory processes. Second, improvements in PPA scores in both intervention groups are due to changes in different functions. SMT showed significant improvements in finger-press reaction time and proprioception, the KIN training group improved significantly in muscle strength and contrast sensitivity.

The improvement in vision following KIN training is consistent with previous research that has found associations between computer game play and visual improvements. For example, Hale et al. [27] have reported that simple computer skills training improved contrast sensitivity in older people and Li et al. [28] found that young expert action video game players had superior contrast sensitivity compared to age- and gender-matched non-action game players. Therefore, navigation through the system menu from a 3 m distance and playing exergames that required central and peripheral vision under changing contrast conditions and accurate, visually guided actions could have resulted in improved vision.

Finally, both exergames emphasised balance training but did not improve postural sway suggesting that participants did not challenge their postural stability at the appropriate intensity and/or duration to improve balance skills. While we acknowledge that other more task-specific measures of dynamic balance might have been more suitable, it should be noted that both trials did not find beneficial effects for other balance measures [*papers under revision*]. A pilot study of the SMT found significant improvements of postural sway after eight weeks of Stepmania training in well-functioning older people [7]. The different finding may be partially explained by a higher proportion of frail people in the current SMT study, suggesting that this type of training might be more suitable for vigorous older people. On the other hand, the current SMT study added games with an emphasis on cognitive tasks resulting in reduced training time of dynamic postural control challenge [29] compared to the Stepmania game training only. As the KIN exergames were adapted from successful training programs, the lack of improvement was most likely due to insufficient exercise time. Therefore, while some differences between the interventions were due to different training content and task-specificity, adherence to the intervention was another major factor impacting on the results. Adherence in both studies was below the recommended doses (SMT 60 min and KIN 120 min per week), and this may have resulted in an insufficient exercise dose to induce benefits. However, despite low adherence rates several improvements were achieved, suggesting a favourable dose-response relationship.

The two exergames used in this study both have advantages and disadvantages. Both systems require significant space in participants’ homes. A major difference between the two systems is the physical mat necessary for SMT. A stepping mat placed on the floor could be viewed as an advantage, because it provides real (tangible) targets for participants during step games. On the other hand, such fixed targets could be seen as a disadvantage as participants have to keep touch with the stance position in order to perform a correct step. Step mats could also present trip hazards and restrict games to those that require stepping. By providing greater flexibility in game designs, step directions and distances, KIN addresses some of these limitations. However, current camera capture abilities are sometimes limited (e.g., bright sun light may disturb sensor capturing). Finally, as the KIN system requires participants to interact with the game through gestures, a greater level of tech-savviness is required.

Both exergame interventions are in initial research and development stages. Therefore, the low exercise adherence can to a large extent be explained by technical difficulties related to the prototype versions of the systems. While individuals frequently reported enjoying playing the games, they often could not manage to start them due to technological issues (bugs), system complexity (multiple components) and usability problems (navigating through the menu). The majority of participants in both interventions required additional visits by research staff to fix technical problems and provide additional training. As part of the studies, participants were offered extensive support which would not be feasible in a real-life setting. These findings therefore suggest the need for substantial system refinement (i.e., reliability and usability) in order to be suitable for older individuals with limited technical experience and skills. Particularly, the development of one-touch solutions is promising to maximise participant acceptability and subsequent adherence and efficacy.

This study had a number of strengths and limitations. A major strength was the comparison of two exergame interventions that were carried out in an unsupervised home setting. It thus provides results of “real world” training and older people’s ability to use exergame technology. The findings that both exergames were safe and improved some aspects of fall risk are encouraging. We also acknowledge certain limitations related to the statistical analysis design. Although considerable efforts were made to use common methodologies across the two studies, logistical issues resulted in study samples that differed with respect to a number of baseline measures. For example, the different minimum age-limit resulted in one participant aged below 70 years in the KIN study. However, the adjustments for baseline test scores and age in the statistical analyses will have controlled for such differences. Sample size differences may have resulted in a greater likelihood of detecting significant differences for SMT, and this appears to be the case for PPA scores where the effect sizes for improvements in both interventions were similar (and significant improvements were evident in the iStoppFalls sample as a whole in preparation). Finally, as indicated above, technological problems contributed to relatively high dropout rates and reductions in training time. Future studies investigating the effectiveness of technology-based intervention programs should include a process evaluation to explore how and to what extent technological problems affect the intervention [30]. However, in this emerging field, improvements to equipment (cameras and step mats), software, internet connectivity and game interfaces are highly feasible. With increasing information technology (IT) awareness among older people, such refinements would present significant scope for acceptable and efficacious training systems in the future.

## Conclusion

The study findings indicate the safe use of exergame interventions in the home setting. Both interventions reduced physiological fall risk while SMT additionally improved central processing speed and specific executive functions. Neither intervention improved balance control. Adherence to both interventions was impacted by technical problems with the exergame systems. Further refinement is required to maximise the benefits and potential that exergames may have for the delivery of home-based fall prevention programs in older people.

### Informed consent

All procedures followed were in accordance with the ethical standards of the responsible committee on human experimentation (institutional and national) and with the Helsinki Declaration of 1975, as revised in 2000 (5). Informed consent was obtained from all patients for being included in the study.
